# Colocating Teleophthalmology Within Primary Care Settings to Improve Access to Diabetic Retinopathy Screening: Retrospective Descriptive Evaluation

**DOI:** 10.2196/17838

**Published:** 2022-10-26

**Authors:** Tiffany Wandy, Shuja Rayaz, Jenna Ashly Levenson Brager, Michael Kiritsy, Elizabeth Offermann, Daniel Durand

**Affiliations:** 1 LifeBridge Health Baltimore, MD United States

**Keywords:** diabetic retinopathy, screening, telemedicine, teleretinopathy, population health, value-based care, quality measure performance, accountable care organization, ACO performance

## Abstract

**Background:**

Annual retinal exams for patients with diabetes are critical as diabetic retinopathy is the number one cause of preventable blindness in working-age adults in the United States. Currently, most patients with diabetes in the United States receive a referral from their primary care provider to see an ophthalmologist for their annual dilated eye exam, which can be an added inconvenience and expense. As such, there is a need for alternative screening strategies within an outpatient network. The use of a telemedicine platform in a primary care setting serves as a novel strategy to increase diabetic retinopathy screening rates. In order to provide better access to diabetic retinopathy screening for our patients, cameras were placed in 3 primary care practices in October 2017 as part of an 8-month pilot program. Specialized cameras from Intelligent Retinal Imaging Systems (IRIS) were used to acquire images that could be interpreted remotely by ophthalmologists within the LifeBridge Health network for the diagnosis of diabetic retinopathy and the detection of other types of pathology (eg, macular edema).

**Objective:**

The aim of this retrospective descriptive study was to examine whether a telemedicine platform can be used as a cost-effective way to increase diabetic retinopathy screening rates in the primary care setting.

**Methods:**

Aggregate screening volume and diagnostic data were collected for each of the 3 practice locations for the 8-month pilot period (October 30, 2017, through June 30, 2018). Additionally, payor reimbursement data and equipment cost data were used to determine the payback period for each of the 3 practice locations.

**Results:**

The pilot program proved the business case that implementation of the IRIS camera in 3 practice locations could result in enough patients being screened to pay for the cost of the camera within a maximum of 2 years. The 3 practices showed increased diabetic retinopathy screening rates of 1%, 6%, and 24%, respectively, and were all able to screen enough patients to be on track to pay off the cost of the camera within 2 years of implementation. Aggregate data from the pilot period showed that of the 1213 patients who were screened, approximately 17.1% (n=207) were diagnosed with diabetic retinopathy and an additional 17.7% (n=215) were suspected of having some other form of pathology. Of note, 10.1% (n=123) were also identified as being “IRIS saves,” defined as having pathology identified that was severe enough to be considered an imminent threat to their vision.

**Conclusions:**

This retrospective descriptive study suggests that a telemedicine platform can be used to improve diabetic retinopathy screening rates in the primary care setting within a large health care system in a cost-effective way that allows for the cost of the equipment to be recouped through billing within a maximum of 2 years.

## Introduction

Diabetic retinopathy is the leading cause of blindness among working-age adults in the United States [1]. Early detection and treatment can prevent or delay blindness due to diabetic retinopathy in 90% of patients with diabetes, but 50% or more of patients do not obtain an eye exam or are diagnosed too late for effective treatment [1]. As a result, early screening is crucial as retinopathy is often asymptomatic but can progress over time to vision loss [2].

The American Diabetes Association recommends an annual eye exam for all adult patients with diabetes [3]. Annual eye exams are rather burdensome in that they involve a referral to an ophthalmologist for a standard, in-person dilated retinal screening each year. A preliminary analysis within our health system suggested that primary care providers are usually diligent in providing patients with referrals to ophthalmologists, but many patients do not follow their primary care provider’s advice and get screened. This lack of follow-through was due to a number of challenges and barriers, including the patient’s lack of understanding of risk, the burdens involved in additional doctor visits for patients who were already under the care of multiple physicians, the costs associated with specialty care, obstacles to accessing care, and the confusing and fragmented nature of the US health care environment.

In recent years, practitioners have looked to alternative methods for eye screening to overcome patient- and system-level barriers. Telemedicine for diabetic retinopathy screening can be advantageous compared to a traditional dilated eye exam because it does not involve dilation of the eye, can usually be done in a short amount of time (total time <15 minutes), does not require a specialist copay cost on behalf of the patient, does not require a specialty provider to acquire the images, and does not require additional travel for patients when the exam is performed in a primary care setting during a regularly scheduled appointment [4,5]. Using telemedicine gives the opportunity for health systems to increase screening rates and provide the proper standard of care for their patients with diabetes.

Intelligent Retinal Imaging Systems (IRIS) is a company that provides a secure platform for capturing and grading retinal images that can be interpreted remotely by an ophthalmologist. The IRIS platform was identified based on the value of the platform, the quality of the retinal images, and the company’s commitment to implementation success (in-person and virtual assistance). In this study, specialized IRIS cameras were used to acquire retinal images at 3 outpatient practices, which were then automatically uploaded to an online platform that served as a data warehouse for these images [6]. The IRIS online platform is currently approved by the US Food and Drug Administration for the diagnosis of diabetic retinopathy and diabetic macular edema [7]. LifeBridge Health primarily used the platform for the diagnosis of diabetic retinopathy, though ophthalmologists interpreting the images at LifeBridge Health’s Krieger Eye Institute were also given the option to identify images for macular edema, glaucoma, macular degeneration, vein occlusion, and hypertensive retinopathy. The objective of this study was to examine the rate of diabetic retinopathy screening with the novel implementation of a telemedicine platform in the primary care clinical setting and show that implementation of the cameras could be cost-effective, even with the small profit margins experienced in primary care.

## Methods

### Study Design

The 8-month pilot program began in late October 2017 with the placement of 2 tabletop TopCon IRIS cameras and one handheld, mobile Volk IRIS camera at 3 primary care practices. Both types of cameras obtain similar quality images, but the handheld unit requires additional training for optimal image acquisition. The tabletop camera costs almost twice as much as the handheld camera but is less subject to damage, for example, from dropping the unit, as can happen with the handheld unit.

The primary care practices included in the pilot were identified using quantitative and qualitative measures. The 3 pilot locations were large, multiprovider practices with a high number of patients with diabetes. Additionally, the practices were enthusiastic about participating in a new technology pilot program, and each identified a physician champion to assist with the implementation. As an indication of the overall patient population, Table 1 describes the insurance payor mix for the 3 pilot primary care practices. Practice #1 is located in suburban Baltimore in Carroll County and includes patients from a mix of payors, with a heavy emphasis (61%) on commercial payor patients. Practice #2 is located in an affluent suburb of Baltimore in Howard County and mainly includes commercial payor and Medicare patients, with no Medicaid. Practice #3 is located in the city of Baltimore and includes the lowest percentage of commercially insured patients.

**Table 1:** Overall insurance payor mix per practice.

Practices	Commercial or private, %	Medicare, %	Medicaid, %	Uninsured, %	Other payor, %
Practice #1	61	15	17	7	0
Practice #2	61	36	0	0	3
Practice #3	43	38	14	5	0

At these practices, patients who had a current diagnosis of diabetes documented in their electronic medical record and who had not had a diabetic retinopathy screening exam documented within the last 12 months were identified during their primary care visits. These patients were then given the opportunity to have a nondilated diabetic retinopathy exam as part of the pilot study. Voluntary participation in the pilot program was offered to all patients who met the inclusion criteria. There were no specific exclusion criteria for this study because the focus of the program was to improve diabetic retinopathy screening rates (specifically whether a patient had a screening or not) among all patients, regardless of payor, in outpatient practices. The diabetic retinopathy screening exam was accepted by all payors in the pilot region, and claims were submitted accordingly for fundus photography with interpretation and report (Current Procedural Terminology [CPT] 92250).

Working with a dedicated LifeBridge Health team, the practices created and implemented workflows, documented processes, and established best practices for camera use in conjunction with primary care physicians and other health care team members at each specific practice. Medical assistants were trained and held responsible for performing the exams and obtaining the images. For each patient screened, 2 fundus images of the retina without the need for dilation were captured via the IRIS cameras within the primary care practices. This is in contrast to the workflow in practices not participating in the pilot where patients were merely referred to an ophthalmologist for a traditional dilated retinal exam, to be performed at a separate visit on another date.

Following the screening, the images were automatically transmitted electronically via the secure online IRIS platform. These images were then read by ophthalmologists at LifeBridge Health’s Krieger Eye Institute via the IRIS platform. Ophthalmologists could manipulate the images using enhancement tools and then conduct a manual diagnostic grading of each image. LifeBridge Health’s Krieger Eye Institute ophthalmologists diagnosed patients as having diabetic retinopathy based on the image review and filled out a templated report to send back to the primary care provider. The ophthalmologists also had the option to designate images as being suspicious for other forms of pathology (including macular edema, glaucoma, macular degeneration, vein occlusion, and hypertensive retinopathy) or flag patients who were identified as having pathology that was severe enough to put them at risk for imminent vision loss for immediate follow-up. If an image was diagnosed as diabetic retinopathy or found to be suspicious for some other form of pathology, the patient was called to schedule an in-person visit with an ophthalmologist for further diagnostics and treatment as needed.

The pre- and postscreening rates for each of the participating practices in the pilot program were obtained by manual chart review. This chart review involved randomly selecting for each provider at least 30 patients with diabetes with office visits in a given month who were eligible for retinopathy screening. The screening rate from the chart review samples was then used to extrapolate the screening rates for the entire practice for all patients with diabetes. The data set for the period from October 2017 to December 2019 used data collected from the IRIS online platform. The IRIS platform provides a dashboard with aggregated data on the number of patients screened, the number of patients diagnosed with diabetic retinopathy or some other form of pathology, and the number of patients who were identified as being at risk for imminent vision loss.

### Data Analysis

The main return-on-investment analysis was performed to determine whether it would be possible for each of the primary care practices to generate enough revenue from screening patients using the IRIS cameras to pay back the cost of the camera within a set period of time. The number of screenings needed to pay off the cost of the camera within 2 years was determined by taking the overall cost of the camera (including shipping and warranty costs) and dividing by the average reimbursement for CPT 99250 for each practice, which varied according to payor mix. The screening goal for each practice was determined by prorating the total number of screenings needed to pay off the camera within 2 years to reflect the 8-month length of the pilot study.

The key clinical analysis focused on whether the diabetic retinopathy screening rates improved with the utilization of IRIS cameras within the 3 pilot practices. This analysis compared the screening rates at the 3 pilot practices before and after the implementation of the IRIS cameras. This analysis compared screening rates from January 2018 to September 2018 to the screening rates at those practices from January 2017 to December 2017, when the practices did not have the cameras or use any type of digital screening (although the pilot was conducted from the end of October 2017 to June 2018, sufficient time was given as a “ramp-up period” for the use of the cameras to be integrated into the practice workflow). Of note, since diagnosis via captured images, compared to in-person examination, depends on the quality of the image, not all images were suitable for diagnosis. The IRIS online platform deemed 88% of all acquired images to be gradable for interpretation by LifeBridge Health’s Krieger Eye Institute ophthalmologists. The analysis presented in this paper includes only those images deemed gradable.

### Ethics Approval

The LifeBridge Health institutional review board (IRB) determined that the study is exempt from ethics approval as it did not meet the definition of human subjects research and did not need a formal review.

## Results

Table 2 shows the total number of screenings conducted during the pilot at each of the 3 practices and breaks down the total screening volumes by the goal (the number of screenings needed to be on track to pay off the cost of the camera within 2 years) and the number of screenings conducted in excess of the goal. All 3 practices screened enough patients to reach their goal, regardless of the type of camera they were using or the payor mix of their patient population.

During the 8-month pilot, practice #1 screened a total of 98 patients. This practice had a handheld camera and needed to screen 30 patients during the pilot to be on track to pay off the cost of the camera within 2 years. Practice #2 screened a total of 164 patients. This practice had a tabletop camera and needed to screen 114 during the pilot to be on track to pay off the cost of the camera within 2 years. Practice #3 screened a total of 196 patients. This practice also had a tabletop camera and needed to screen 138 patients to pay off the cost of the camera within 2 years. The number of screenings needing to be performed to pay off the cost of the camera within 2 years depended on the type of camera the practice had (the tabletop camera costs nearly twice as much as the handheld version) and the average reimbursement based upon payor mix. For context, the average reimbursement across all 3 practices and across all payors was US $56.45, with commercial payors tending to reimburse at the highest rates, followed by Medicare, with Medicaid reimbursing at the lowest rate.

Table 3 depicts the screening rates before and after the initiation of the pilot program at each of the 3 practices, as well as the corresponding percentage change. The 3 practices showed increased diabetic retinopathy screening rates of 1%, 6%, and 24%, respectively.

In total, 1213 patients were screened for diabetic retinopathy. Of these 1213 patients, approximately 17.1% (n=207) were diagnosed with diabetic retinopathy and 17.7% (n=215) were suspected of having some other form of pathology. In addition, 10.1% (n=123) were suspected of having pathology that was deemed serious enough to put them at risk for imminent vision loss. For all patients requiring follow-up (those with any form of pathology), direct referrals were made to an in-network ophthalmologist at the Krieger Eye Institute for further evaluation and treatment. Table 3 also shows the impact of the telemedicine initiative and the improved screening rates achieved at the 3 primary care practices. Practice #3 showed a particularly large improvement in screening rate (24%), most likely due to a best-practice workflow that was implemented at the practice location, specifically one in which medical assistants were able to seamlessly identify and direct patients with diabetes to obtain a retinopathy exam via a standing order.

**Table 2:** Volume of screenings by practice (October 30, 2017, to June 30, 2018).

	Goal (screenings needed to pay off camera in 2 years), n	Screenings in excess of goal, n	Total screenings conducted during pilot, N
Practice #1	30	68	98
Practice #2	114	50	164
Practice #3	138	58	196

**Table 3:** Screening rates before and after implementation of Intelligent Retinal Imaging Systems cameras in the 3 pilot practices.

Practices	Screening rate before pilot^a^, %	Screening rate during pilot^b^, %	Percentage change
Practice #1	39	40	+1
Practice #2	32	38	+6
Practice #3	40	64	+24

^a^Data calculated from January 2017 to December 2017.

^b^Data calculated from January 2018 to September 2018.

## Discussion

### Principal Findings

Our study shows that the implementation of a telemedicine platform for diabetic retinopathy screening in primary care practices can increase screening rates among adult patients with diabetes and be feasible within a primary care environment with low profit margins. Furthermore, integrating camera utilization within the provider workflow allowed for better adoption of its use and therefore increased opportunities for screening patients. Of the 1213 patients who underwent screening, 17.1% (207 patients) were diagnosed as having diabetic retinopathy and 10.1% (123 patients) were found to be at risk for imminent vision loss. The 123 patients at risk for imminent vision loss were subsequently contacted for an urgent follow-up visit with an ophthalmologist. Thus, there was a direct and meaningful impact on patient care due to the implementation of the IRIS cameras and use of the online platform within the LifeBridge Health network.

Diabetic retinopathy is the most common and serious ocular complication of diabetes and is one of the leading causes of vision loss in many developed countries [2,8,9]. Screening for diabetic retinopathy traditionally involves an in-person dilated eye exam with an ophthalmologist; however, even with support from governmental and nongovernmental agencies, medical societies, and various global organizations, a large portion of patients with diabetes do not receive the recommended annual eye exam [10,11]. Telemedicine provides an alternative method for diabetic retinopathy screening and has been shown to be effective for the diagnosis of new diabetic retinopathy as well as other ophthalmologic diseases [12,13]. A large-scale retrospective study involving over 15,000 patients conducted in the United States showed that the use of the IRIS cameras and platform yielded high sensitivity and a low false-negative rate for the diagnosis of diabetic retinopathy [14].

From a population health standpoint, current literature has examined several patient-level barriers to retinopathy screening, including patients having competing priorities, anxiety about the screening, disengagement with diabetes care, misinformation about the screening, and forgetting to attend the screening [15]. Using telemedicine can help curb some of these issues and increase access to care. Patients can have their screening performed during a regular office visit with their primary care physician, eliminating the need for an additional appointment with an ophthalmologist. Retinal images can be interpreted remotely by an ophthalmologist; if necessary, patients can be contacted by a specialist for further evaluation. This workflow directly involves the specialist in the patient’s care after their primary care visit, rather than placing the responsibility on the patient to follow up with the specialist. A multicenter randomized controlled trial conducted over 5 years in the United Kingdom also showed that patients who received digital diabetic retinopathy screening at a primary care office were more likely to receive diabetic retinopathy screening compared to those who underwent screening at an additional office visit with an eye specialist [16]. This finding implies that the use of telemedicine can increase access and adherence to screening, which is particularly important for those patients in lower socioeconomic communities who tend to have a higher prevalence of diabetes [17]. Additionally, this form of retinopathy disproportionately affects the vision of lower-income households, for whom the subsequent disability can be economically devastating. In this study, practice #3, which has the highest percentage of patients with government-subsidized (Medicare and Medicaid) insurance or no insurance and the highest percentage of patients of lower socioeconomic status, showed the highest increase in screening rates after the implementation of the IRIS cameras among the pilot practices (Table 3).

### Strengths

This study is retrospective and descriptive in nature as it examines the novel deployment of a telemedicine system within an outpatient primary care clinical practice network in a major metropolitan area. The original intent of the pilot program was to improve access to diabetic retinopathy screening among our patient population. Overall, we have been able to screen over 1200 patients, many of whom may have not been screened otherwise. It is our view that the implementation of the IRIS cameras within the 3 practices provided a convenient alternative for our patients within the community. Furthermore, 123 out of 1213 (10%) screened patients were found to be at risk for imminent vision loss, and we were able to swiftly identify those patients and refer them to our ophthalmologists in a timely manner for urgent follow-up. Due to the successful implementation in the pilot practices, we have continued to expand the program to additional practices within our primary care network. Since the conclusion of the pilot study, we have conducted a full electronic medical record integration project to ensure an even smoother clinical process for conducting diabetic retinopathic screening and sending clinical grading back to the primary care providers. This initial study provides the foundation for future work, including a possible randomized controlled trial to better elucidate the impact of a telemedicine system in the outpatient primary care setting.

### Limitations

This study was not able to report statistical comparisons or inferences due to a small sample size. However, we were able to identify a clinical benefit with the implementation and use of the IRIS cameras in the outpatient practices as evidenced by the aggregate data mentioned in the Results section. System-level limitations exist when using a telemedicine platform for diabetic retinopathy screening and were shown to affect this pilot study. For example, the use of the cameras requires additional time and effort by the medical assistants in the primary care practices, which can be challenging to operationalize in busy outpatient practices. Though this study was small and had its limitations, the overall results highlight a noteworthy improvement in screening rates with the potential to impact a larger population should this process be rolled out to additional primary care practice locations.

### Conclusions

This study suggests that the use of a telemedicine platform in the primary care setting can be an effective alternative to dilated eye exams performed by ophthalmologists for adult patients with diabetes. Implementation of a telemedicine platform allowed our primary care providers to offer comprehensive care to their patients with diabetes, reducing the need for multiple appointments and specialist co-pays. Our most successful implementation of the telemedicine platform was in the practice with the highest percentage of patients of lower socioeconomic status and government-subsidized insurance or no insurance (practice #3). Telemedicine can provide increased access to care for this type of patient population in particular. Timely screening for retinopathy also had a direct benefit on the clinical care of our patients. Since the implementation of the telemedicine platform, 123 patients were found to be at risk for imminent vision loss and were immediately referred to an ophthalmologist for time-sensitive treatment. Overall, this approach of using medical devices and specialized software to increase screening rates will allow us greater opportunities to provide sight-saving treatments and help to prevent blindness in our patients.

## Abbreviations

CPT: Current Procedural Terminology
IRIS: Intelligent Retinal Imaging Systems
